# Knowledge Graph-Enabled Text-Based Automatic Personality Prediction

**DOI:** 10.1155/2022/3732351

**Published:** 2022-06-20

**Authors:** Majid Ramezani, Mohammad-Reza Feizi-Derakhshi, Mohammad-Ali Balafar

**Affiliations:** ^1^Computerized Intelligence Systems Laboratory, Department of Computer Engineering, Faculty of Electrical and Computer Engineering, University of Tabriz, Tabriz, Iran; ^2^Department of Computer Engineering, Faculty of Electrical and Computer Engineering, University of Tabriz, Tabriz, Iran

## Abstract

How people think, feel, and behave primarily is a representation of their personality characteristics. By being conscious of the personality characteristics of individuals whom we are dealing with or deciding to deal with, one can competently ameliorate the relationship, regardless of its type. With the rise of Internet-based communication infrastructures (social networks, forums, etc.), a considerable amount of human communications takes place there. The most prominent tool in such communications is the language in written and spoken form that adroitly encodes all those essential personality characteristics of individuals. Text-based Automatic Personality Prediction (APP) is the automated forecasting of the personality of individuals based on the generated/exchanged text contents. This paper presents a novel knowledge graph-enabled approach to text-based APP that relies on the Big Five personality traits. To this end, given a text, a knowledge graph, which is a set of interlinked descriptions of concepts, was built by matching the input text's concepts with DBpedia knowledge base entries. Then, due to achieving a more powerful representation, the graph was enriched with the DBpedia ontology, NRC Emotion Intensity Lexicon, and MRC psycholinguistic database information. Afterwards, the knowledge graph, which is now a knowledgeable alternative for the input text, was embedded to yield an embedding matrix. Finally, to perform personality predictions, the resulting embedding matrix was fed to four suggested deep learning models independently, which are based on convolutional neural network (CNN), simple recurrent neural network (RNN), long short-term memory (LSTM), and bidirectional long short-term memory (BiLSTM). The results indicated considerable improvements in prediction accuracies in all of the suggested classifiers.

## 1. Introduction 

Personality is the enduring set of traits and styles that an individual exhibits, that is, those characteristics that represent his/her dispositions, namely, natural tendencies or personal inclinations [1]. Being aware of the personality characteristics of people will help them improve their relationship management skills and also ameliorate their interpersonal communications, regardless of the type of relationship, as it happens between two friends, the boss and employee, investor and investee, seller and buyer, between members of a family, and so on.

With the advent of social networks and their remarkable fortune among people, nowadays, a great deal of communication happens through social networks. Language, as the main communication tool among humans that competently represents their thoughts, emotions, opinions, and totally personality, is also used in written and spoken form among social networks' users to communicate with each other. Admittedly, having some information about the personality with whom you are communicating would be so advantageous. It can be carried out by analyzing the exchanged texts (which is also known as written language), among the users of such information infrastructures. Accordingly, the automatic prediction of human personality through computational approaches is called Automatic Personality Prediction (APP).

What we know about text-based APP is largely based upon empirical studies that have investigated how to exploit different methodologies for the purpose of personality prediction of individuals in Internet-based infrastructures (like social networks). Actually, various hypotheses regarding this issue can be found that they are commonly concerned about achieving a more knowledgeable substitutions for text elements to deal with, rather than pure strings of characters.

In the history of text-based APP, initial investigations have mostly focused on linguistic features of text elements to achieve more knowing about them [2–5]. Over the years, it has received much attention; while some studies have applied a combination of linguistic features and machine learning methods [6–9], some others have focused solely on the machine (deep) learning methods [10–13]. In the last few years, we have witnessed a considerable rise in text-based APP, which have used embedding methods to transfer the text elements to a more meaningful space (rather than character space), in favor of better exploitation of computational methods [14–17].

Generally, it can be inferred that all of the investigations are intended to acquire more knowing about the text elements, each of which is done through applying miscellaneous methods. Indeed, they are absolutely right; namely, the knowing will be the basis of predictions.

Although various researches have been carried out on text-based APP, no study has been found that is essentially intended to focus on Knowledge Representation (KR). This paper for the first time (to the best of our knowledge) calls into question the application of knowledge representation and thereby knowledge graph in text-based APP. Specifically, this study makes a major contribution to research on automatic personality prediction by proposing a novel knowledge graph-enabled system. Indeed, it meticulously investigates knowledge representation as a novel solution for text-based personality assessment. We believe that there is knowledge, and then there is knowing. Therefore, at first, we should discover the world behind the words. It will provide an important opportunity to advance the understanding of text elements. In consequence, practically, the significance of our method is that it empowers an APP system to achieve a comprehensive representation of the appearing concepts in the input text, which entails the knowledge behind them and models the semantic relations among them, in a more comprehensible manner for the machine, as the basis of its predictions. In fact, the proposed method equips machine with the required knowledge to acquire a better understanding of the entailed concepts in the input text and accordingly achieve better results.

Knowledge representation is a field of artificial intelligence dedicated to representing information about the world in a form that a computer system can utilize to solve complex tasks [18]. In fact, knowledge representation is necessary to understand the nature of intelligence and cognition of concepts so well that computers can be made to exhibit human-like abilities [19]. Therefore, in the case of expecting human-like abilities from artificial intelligence, it seems that we ought to represent the knowledge of the world for it. Meanwhile, the knowledge graph is actually the outcome of knowledge representation. It organizes the knowledge of concepts in a graph structure and integrates all existing information about them.

Therefore, aimed to design a knowledge graph-enabled text-based APP system, this paper proposes a three-phase approach that includes:

- Phase 1: preprocessing that contains four steps of needed preprocessing, that is, tokenization, noise removal, normalization, and named entity recognition to make the input text ready for main processes in next phase.
- Phase 2: knowledge representation, the main contribution of this study that comprises three steps, that is, graph building, graph enriching, and graph embedding. In practice, this phase first attempts to build the corresponding knowledge graph for a given text, which is a knowledgeable representation of the input text and then enriches it to cover some neglected pieces of knowledge about concepts. At last, the acquired enriched graph is embedded to a more computationally applicable space, to facilitate the computations in next phase.
- Phase 3: automatic personality prediction that aims to predict the personality traits for each input text through a multilabel classification model. To do so, four base deep learning models were proposed that includes Convolutional Neural Network- (CNN-) based, simple Recurrent Neural Network- (RNN-) based, unidirectional Long Short-Term Memory- (LSTM-) based, and Bidirectional Long Short-Term Memory- (BiLSTM-) based classifiers.
- This study aimed to address the following research questions:
- RQ.1: How does knowledge graph enabling influence the performance of a text-based automatic personality prediction system?
- RQ.2: What are the performances of popular deep learning models, including CNN, simple RNN, LSTM, and BiLSTM in multilabel classification of knowledge graphs' embedding matrices? Which of them outperforms the others?
- RQ.3: Does knowledge graph enabling of an APP system affect equally the predictions in all five personality traits in Big Five model?

The remaining part of the paper proceeds as follows: Section 2 is concerned with the Big Five personality model.  Section 3 provides an overview of text-based APP systems. The proposed knowledge representation-based APP system is meticulously demonstrated in Section 4.  Section 5 presents the findings of this study, and then Section 6 includes a discussion of the implication of the findings as well as responses to the research questions. Finally, Section 7, namely, the conclusion, gives a brief summary and critique of the findings.

## 2. The Big Five Personality Model

So far, various personality trait models have been introduced [20]. In this study, the Big Five model (Five Factor Model) [21], as the most widely accepted trait model that capably correlates with human traits that are presented in written language [22], is used. It basically demonstrates the individuals' personality in five categories: openness, conscientiousness, extroversion, agreeableness, and neuroticism. OCEAN is the acronym of the five categories, which we shall refer to, also. Each of the five personality trait represents a range between two extremes [23]; that is, extroversion represents a continuum between extreme extroversion and extreme introversion. To make it more clear, indicating some facets of each trait, which occurs in those people with high scores for each trait, may be useful (for more details, please refer to [24, 25]):

- Openness (O): an inclination to embrace new ideas, arts, feelings, and behaviors; unconventional; focused on tackling new challenge; wide range of interests and so imaginative.
- Conscientiousness (C): an inclination to be so self-disciplined, well organized and dutiful; careful and hard-working; reliable, resourceful and on time.
- Extroversion (E): an inclination to be outgoing, energetic, assertive and talkative; affectionate, sociable and articulate; enjoys being the center of attention.
- Agreeableness (A): an inclination to agree and accompany the others; altruist and unselfish; friendly, loyal and patient; modest, considerate and cheerful.
- Neuroticism (N): an inclination to experience negative emotions like anxiety, anger, depression, sadness, and envy; impulsive and moody; lack of confidence.

Furthermore, it is worth noting that the Big Five traits are mostly independent [23]. It means that being cognizant of someone's one personality trait does not provide so much information on the remaining traits of the Big Five model.

## 3. Literature Review

In recent years, there has been an increasing amount of literature on APP that mainly pays particular attention to predict the personality from text, speech, image, video, and social media activities (likes, visits, mentions, digital footprints, profile interpretation, etc.).

Text as an appearance of human language would competently reflect the writer's personality [22]. Due to this fact, it is always a matter of concern for personality psychologists. Spreading the Internet-based communication infrastructures increased the text-based communications among people. It opens the door for computational psychologists to investigate the personality of writers from exchanged texts. Here, we will review the researches conducted on text-based APP.

Taking a glimpse into the reported investigations in APP, it can be claimed that, generally, all of them are intended to acquire more meaningful and knowing-full alternatives to input text's elements (namely, words, terms, or, generally, all the appearing concepts) to deal with. In simple words, dealing with more meaningful alternatives that convey more knowing and information rather than pure character strings is highly preferred. Actually, this knowing about written language elements may better represent the knowledge behind them and may lead better predictions about writer's personality. Tracing the evolution of text-based APP systems sheds more light on this claim. With respect to this claim, generally, we can classify the previous studies into five categories: lexicon-based methods, hybrid methods (combination of lexicon-based and deep learning-based methods), embedding methods, ensemble modeling methods, and network-based methods. A detailed analysis of these categories is given bellow.

### 3.1. Lexicon-Based Methods [2–5, 26]

Rudimentary techniques have mainly tended to utilize lexicons, which provide linguistic and statistical knowing about text elements. Lexicon-based methods primarily try to predict the personality of writer through assigning his/her words to predetermined categories. Linguistic Inquiry and Word Count (LIWC) [27] is one of the most common tools that counts words in psychologically meaningful categories and calculates the degree to which people use different categories of words. It is simply a dictionary of words and word stems, each of them belonging to a one or more category. Given a text, LIWC calculates the percentage of included words in each category. The main idea behind LIWC is that the word usage in everyday language reveals the thoughts, personality, and feeling of individuals. There have been different versions available since 2001. Further information is available at https://liwc.wpengine.com/ and there are more than 80 categories in LIWC2015. Mairesse features [2] and Structured Programming for Linguistic Cue Extraction (SPLICE) are other options that provide linguistic features for words.

In their analysis of APP from the words that people use, Yuan et al. [5] have investigated the personality of the characters in vernacular novels. They have created a vector for each dialog using LIWC features, which reflects the psychology of the characters. Finally, the vectors have been mapped to the Big Five personality traits, to predict the final personality labels. Mairesse et al. [2] have also investigated a miscellaneous variety of lexicon-based features in order to predict the Big Five personality traits from written text and spoken conversation.

Among the first reports on APP from the social media text, Golbeck et al. [3] have considered LIWC features over the 167 samples of Facebook text contents as well as the users' profile information. The results confirmed limited improvements in APP. In the same manner, the authors in [4] have studied the Big Five personality traits from Twitter posts besides the users' profile attributes. They have actually intended to find antisocial traits of narcissism, Machiavellians, and psychopathy (commonly referred to as the dark triad) through using LIWC features. Perusing the reported results implies that prediction of personality traits from social media text using lexicon-based methods could not considerably improve the APP accuracy.

Later, in a study that has been set out to predict personality traits from social networks' microblogs, Han et al. [26] have found that the context-based knowledge of words may be advantageous to personality prediction. They have believed that since the traditional psychological lexicons (like LIWC) are appropriate for formal texts, they could not efficiently be applied in social networks' informal texts. Therefore, they have proposed an approach to automatically extract a personality lexicon from social networks, through using keyword extraction techniques and then semantically clustering the extracted keywords. At last, they have simply combined the extracted lexicon (as a prior-knowledge source) with the word embedding vectors and have fed them into a classification model, to predict the Big Five's personality traits' labels. They have partially enhanced the prediction accuracy, even though they have just taken the advantage of words' lexical knowledge.

### 3.2. Hybrid Methods [6, 9, 11, 28]

Generally, in the literature, there seems to be no a tendency among researchers to use lexicon-based methods, solely. Telling the truth, it is hardly fair to shift all the APP responsibility solely to them, because of their superficial knowledge of text elements. Consequently, a large and growing body of literature has investigated the combination of lexicon-based methods with more knowing-full methods that fairly has improved proportionally the predictions' accuracy.

Designing a convolution neural network (CNN), which uses the document-level Mairesse features (extracted from the input text) in an inner layer, has formed the central focus of a study by Majumder et al. [6]. They have trained a separate identical binary classifier for each of the five personality traits in the Big Five model that receives sentences of the input text one by one and then aggregates them into a document level vector. Besides, they have finally ignored all the emotionally neutral sentences, to improve the performance. Yuan et al. [9] have carried out a study to predict the personality of users from their Facebook status contents. Actually, they have combined the LIWC features with deeper features that have been extracted through a deep learning model. They firstly have extracted the language features via the LIWC tool, and then using a CNN, they have automatically extracted the features from textual contents. Subsequently, the two extracted features have been combined to predict the personality labels.

In another investigation into APP from texts in online social networks, the authors in [11] have proposed a bidirectional LSTM model, called 2CLSTM. In order to detect user's personality using the structures of texts, the model has been strengthened by a CNN as well as a latent sentence grouping module, which has been applied to capture closely connected sentences. Xue et al. [28] studied the effects of semantic representation of words in APP systems. They acquired a word-level semantic representation of text elements and then fed them into a neural network to obtain higher-level semantics of text elements.

### 3.3. Embedding Methods [14–17, 29–31]

Alongside these researches, many attempts have been made with the purpose of utilizing complex methods that use even more knowing-full alternatives for text elements. Indeed, they have succeeded in achieving better results during predictions. They mostly pay particular attention to embedding methods, which transform the text elements from a textual space to a real-valued vector space. Overall, these methods, despite their variety, have better performance in APP, rather than previously mentioned methods. This ability is a consequence of embedding methods' adroitness in meaning acquisition and representation. In a study that has been set out to detect personality based on text content analysis, Ren et al. [14] have investigated a novel multilabel personality prediction learning model, which combines emotional and semantic features. In particular, they have leveraged a Bidirectional Encoder Representation from Transformers (BERT), to generate sentence-level embeddings for extracting semantic features from text, as well as a sentiment dictionary for the sake of text sentiment analysis purposes. Encoders primarily are designed for achieving a knowing-full representation of input text. They have used the Myers-Briggs Type Indicator (MBTI) and Big Five personality trait models in their study. Xue et al. [15] have also designed a deep learning-based method for personality prediction from text, which are posted in online social networks. They have recommended AttRCNN, a hierarchical model that uses a sentence-level encoder that is followed by a document level encoder in order to achieve the deep semantic features of text posts. Moreover, they have concatenated the deep semantic features with the statistical linguistic features obtained directly from the text posts and have fed them into a regression model to predict the Big Five personality traits' labels. Exploiting the embedding methods abilities, in their study, Christian et al. [17] have suggested a multimodel deep learning architecture for personality prediction, which was combined with various pretrained language model including BERT, RoBERTa, and XLNet as a feature extraction method on social media text. The main idea behind their investigations was that since the common deep learning models such as recurrent neural networks (RNNs) and LSTMs suffer from some drawbacks that are defeated using embedding methods, the embedding methods practically outperform them. Specifically, they mostly suffer long training times and inability to capture the context-based information of words and thereby the true meaning of words. At last, the final predictions have been taken based on averaging the output of different pretrained models. Other researches ([16, 29–31]) have also investigated designing embedding-based APP models that make predictions from text.

### 3.4. Ensemble Modeling Methods [13, 24, 32–34]

Meanwhile, taking the advantages of several classifiers and benefit their prediction abilities simultaneously was a matter of concerns for some studies. Utilizing different APP models predictions, the authors in [24] have proposed an ensemble modeling method. Specifically, they have suggested five separate APP models, including term frequency vector-based, ontology-based, enriched ontology-based, latent semantic analysis-based, and deep learning-based (BiLSTM) methods. Then, all of the individual five models have been gathered through a Hierarchical Attention Network (HAN) as the meta-model. In consequence, they have benefited the ability of five distinct APP model, to make the final decisions about Big Five personality traits. In their study, El-Demerdash et al. [13] have suggested a transfer learning-based APP method that have got the benefits of leading pretrained language models such as Elmo, ULMFiT, and BERT. To raise the overall personality prediction performance, they have applied a model consists of fusion strategies on data level and classifier level. Adopting the tree pretrained models, they have used the fusion of Essays and my personality datasets for further fine-tuning of the proposed models. Using independent classifiers, each model performs APP separately. Then, the results have been fed into an ensemble learning model that combines multiple classifiers' outputs, to acquire more reliable prediction. Having the same objectives, other researchers [32–34] have questioned the usefulness of such an approach.

### 3.5. Network-Based Methods [35, 36]

There are also a number of investigations that have aimed for achieving a different representation. They mainly have focused on modeling the network among the online social media users. The first report on group-level personality prediction was conducted by Sun et al. [35]. They have proposed an unsupervised feature learning method called AdaWalk that takes the advantage of independence from labeled dataset. Actually, it was designed based on Network Representation Learning (NRL) method, which was suggested by the authors. Practically, it constructs a complete graph in which its vertices are the users. The graph also possesses the generated texts for each user, the similarity between each users' texts, and the personality labels in Big Five model. Subsequently, applying random walks (AdaWalks) on the graph, they have transformed the network to a set of sequences and finally have predicted their personality labels after embedding all of them. In the same vein, Guan et al. [36] have suggested personality2vec, which predicts the personality labels based on NRL using online social networks' texts. The authors have intended to fully utilize the semantic, personality based, and structural information of user generated texts.

Regarding the evolution, the aforementioned claim that all of the contributions have been attempted to achieve more meaningful alternatives for text elements to deal with would thus seem to be defensible. Actually, the contributions provide strong experimental evidences that more knowing-full alternatives for text elements may lead more reliable results. What is not yet clear is the impact of an approach that is fundamentally based on knowledge representation of text elements on APP. An approach that provides really knowledgeable alternatives for text elements conveys all of the related information and knowing about the concepts as well as their relations.

## 4. Material and Methods

The purpose of knowledge representation approach is to demonstrate the cognitive perceptions behind the key concepts in the world, as well as the relations among them. The dexterity of intelligent functionality is remarkably correlated with existed represented knowledge, both for human and seemingly for machine. We thus primarily decided to represent the knowledge behind the input text elements in favor of APP objectives. To do so, it was decided to manipulate RDF modeling. The aforementioned abilities of RDF model justify its competency in knowledge representation.

### 4.1. Dataset and Some Statistics about It

In this study, the provided essays in Essays Dataset [37] were used for training and testing the proposed APP model. It consists of 2,467 essays, which are written by psychology students. Afterwards, they were asked to fill out the Big Five Inventory Questionnaire. At the end for each essay, a binary label was assigned to each five personality traits. Throughout this paper, each individual essay will be referred to as text. Moreover, it should also be noted that the Big Five personality model was used all over the investigations.

**Figure 1:**
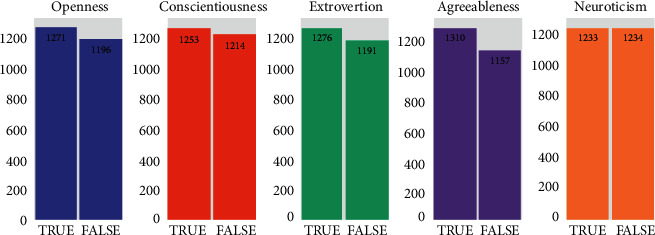
The distribution of labels in each five personality traits in Essays Dataset.

Let us scrutinize much more information about the Essays Dataset.  Figure 1 depicts the distribution of True and False labels throughout the dataset individually in each of the five personality traits. Slight difference between the number of True and False labeled essays reveals that the dataset is balanced and appropriate for learning the APP model.

**Figure 2:**
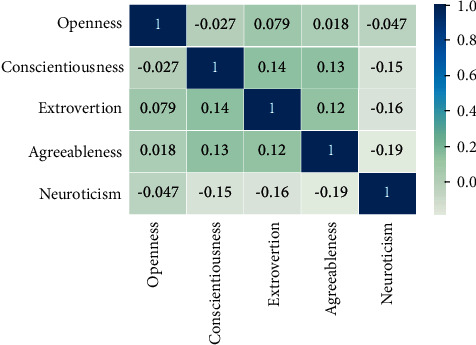
The correlation matrix for five personality traits in Essays Dataset.

Figure 2 compares the correlations among the five personality traits in Essays Dataset. As it can be seen, a correlation matrix is a symmetric matrix, in which all the values on the main diagonal are equal to 1. The correlation coefficient can range between −1 and +1. The larger the absolute value of coefficient, the stronger the relationship between two traits. Specifically, a positive coefficient between two traits means that being aware of one trait's label allows a correct prediction of the other; as close as possible to +1, it will conclude more correct predictions.

**Figure 3:**
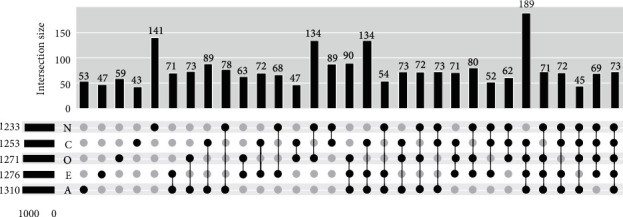
The UpSet plot of intersections among true labeled sets of personality traits in Essays Dataset. Notes: (i) sets {O, C, E, A, N} are sorted by their cardinality in ascending order; (ii) light (empty) circles indicate that the set is not part of that intersection.

The UpSet [38] plot of five sets of personality traits is presented in Figure 3. An UpSet plot actually is considered as a substitution for Venn diagram, when dealing with more than 3 sets. Having five sets of personality traits (namely, O, C, E, A, and N), the UpSet plot makes it possible to provide an efficient way to visualize the intersections of five sets. Each row at the bottom of Figure 3 denotes to a set, and each column corresponds to one segment in Venn diagram, depicted with five light or black circles. A black circle indicates that the corresponding set is participating in the intersection, and a light circle vice versa. Indeed, a light circle indicates that the complement of the set (O′, C′, E′, A′, or N′) is participating in the intersection. In particular, the rightmost column that has five black circle for all of the five sets is equal to (O ∩ C ∩ E ∩ A ∩ N). The bar chart on top of Figure 3 represents the cardinality of each corresponding intersection. It worth mentioning that the plot depicts the intersections among true labeled essays' sets. That is to say, just the true labeled essays in OCEAN traits are taken into consideration.

### 4.2. System Architecture

**Figure 4:**
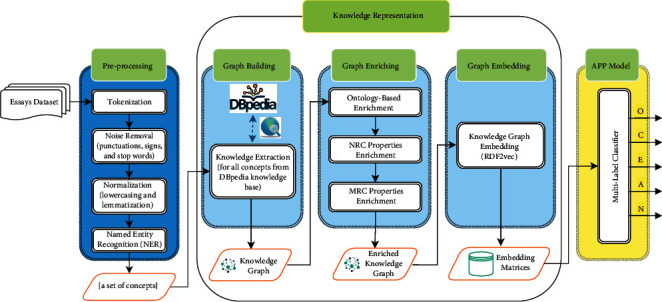
System architecture.

Aimed to answer the research questions stated at the beginning of this study, we suggested a three-phase approach, which is outlined in Figure 4. The experiment proceeds with the following phases below.

#### 4.2.1. Phase 1: Preprocessing

In this phase, the aim was to clean and transform the input texts into a more digestible form for machine to be processed in next phase. This traditional prominent and common practice in natural language processing basically consists of miscellaneous activities depending on the existing task. What follows is a description of preprocessing activities that were carried out in first phase, as depicted in Figure 4.

*(i) Tokenization*. Having a text, “tokenization” is the task of chopping it up into pieces called tokens, which roughly correspond to words [39]. Tokens are also deemed as the smallest useful semantic unit for processing. For this purpose, the tokenizer, which is provided by Natural Language Toolkit (NLTK) [40], was used.

*(ii) Noise Removal*. In the interest of achieving more plain text, it was necessary to remove undesirable and interfering pieces of input text. Regarding the current task, we removed punctuations, signs, and stop words using NLTK.

*(iii) Normalization*. “Normalization” is the process of canonicalizing tokens to a more uniform sequence, so that matches occur despite superficial differences in the character sequences of the tokens [39]. It practically decreases the amount of information that the machine has to deal with, those that are conceptually similar, but morphologically different. In an attempt to normalize the input text, lowercasing and lemmatization were carried out.

Lemmatization is the morphological analysis of words that groups together their inflected forms and returns their bases or dictionary forms, which are called lemma. Since lemmatization converts words to their meaningful dictionary form and yields the correct form of concepts, which really exist in the world compared to stemming, which is an alternative method to reduce inflected words to their stems and usually is fulfilled through chopping off the ending characters of the word, it usually returns incorrect and misspelled forms of words, and it is appropriate in the current task. The resulting meaningful concepts will be queried during knowledge graph building in the following phase. In this study, the lemmatization was also carried out using NLTK.

*(iv) Named Entity Recognition (NER)*. With an eye to achieving the knowledge behind the words, it will be necessary to recognize the named entities from the input text. The sequences of words are actually the name of things (that is to say, the name of organization, person, company, event, etc.). As a matter of fact, they convey more information than do the other words. In current study, the spaCy NER [41] was used to recognize named entities.

After the completion of preprocessing phase, what is extant constitutes a set of concepts, which convey the fundamental notions that have appeared in the input text. It should be mentioned that, after the NER, duplicate elements were removed from the set of concepts. Then, in order to prepare the elements to be matched with DBpedia knowledge base entries, first letter capitalization was performed for all of the elements, and white space replacement with underscore was done for multiword elements as well. Now, everything is ready to find out the world behind the words.

A brief summary of the phase 1, as it can be seen in Figure 4, may be described as follows:

- Input: essays' texts from Essays Dataset;
- Output: a set of extracted concepts for each text;
- Objective: to prepare a more digestible form of input text for main processes in next phases.

#### 4.2.2. Phase 2: Knowledge Representation

As it was stated in Introduction, we chose the graph structure to represent the existing knowledge of concepts in the input text, as well as the relations among them, and to eventuate a knowledge graph for each text. The two first steps in bellow fully describe how a knowledge graph was built for each text. The purpose of the current phase is to attain a comprehensive representation of existing knowledge of the input set of concepts, so that it could be applied for subsequent computations. Hence, the resulting knowledge graph was transferred to a numerical space using a graph embedding method in third step. The suggested three-step procedure is shown in Figure 4 and proceeds as follows.

**Step 1 .**

Knowledge Graph Building

As a matter of fact, the set of extracted concepts from the input text in phase 1 substantially organizes the existent notions in it. There is always knowledge behind every concept. The current step is intended to extract the knowledge of appeared concepts in input text from DBpedia knowledge base [42] and then tries to establish a knowledge graph, which effectively organizes and represents the knowledge of containing text elements [43].

A knowledge graph is a large-scale knowledge base composed of a large number of entities (objects, events, or concepts) and relationships between them [44]. Actually, it is a directed heterogeneous (having vertices/edges of different types) labeled multigraph (a graph, which is allowed to have multiple directed edges between the same pair of vertices), in which the labels have well-defined meanings [45]. The graph structure in knowledge graph adroitly possesses what is needed in knowledge representation [46]. Like all graphs, it consists of vertices and edges, in which the vertices represent the entities of real world, and the edges connect pairs of vertices according to their relationship. What is more, the labels convey the exact information (sometimes called semantics) about the existing relationship (edge) between the vertices. The encompassed knowledge in the knowledge graphs is stored in the form of triples same as (*h*, *r*, *t*) that stands for (head entity, relationship, tail entity). That is to say, having a set of vertices *V*, along with a set of labels *L*, the knowledge graph would be a subset of the cross product *V* × *L* × *V*; each member of this set is referred to as a triple [45]. Each triple may also be interpreted as (subject, predicate, object); for instance (Louvre, is located, Paris). [47] provides detailed information about knowledge graphs.

Meanwhile, there is a well-suited framework that matches as close as possible the knowledge graph's triple requirement, namely, Resource Description Framework or RDF. In essence, it is a standard for representing information in the Web. Equally, this framework is made up of (subject, predicate, object) triples. A set of RDF triples that constructs an RDF dataset can be also viewed as a directed heterogeneous labeled multigraph (like a knowledge graph), which is also referred to as RDF graph [48]. In an RDF graph, vertices (subjects and objects) are either Internationalized Resource Identifiers (IRIs), which stands for a Unicode string representing resources, or literals that contain values such as strings, numbers, and dates. Also, the edges (predicates or labels) are also IRIs representing predicates or relationships. More detailed information about RDFs is available at https://www.w3.org/TR/rdf11-concepts/.

Intended to build the knowledge graph of input text during graph building step in phase 2, as previously mentioned, the DBpedia knowledge base is used. DBpedia actually is a community effort to extract structured information from Wikipedia and to make them available on the Web. The 2016–04 release of the DBpedia contains 9.5 billion RDF triples that describe about 6 million entities.

One can easily query on DBpedia dataset online via SPARQL endpoint, which is a standard query language and protocol for linked open data and RDF databases. Thus, we queried all the elements of input concepts' set on DBpedia and extracted all the relevant knowledge of each concept. It was fulfilled through “DESCRIBE” in SPARQL query language (with no binding in SELECT clause and no pattern in WHERE). Specifically, it asks for a description about queried concept (sometimes called resource) and receives any concepts or resources, which are directly related to the queried concept (for further details about SPARQL query language, please refer to [49]). As previously mentioned in Section 1, the results returned from the queries are in the form of RDF triples that provide a set, which is also called RDF graph. RDF graph organizes the knowledge of concepts in a directed heterogeneous labeled multigraph, which is wildly known as knowledge graph. It almost encompasses all the (existent) knowledge of concepts. One can find the results for a given query X on DBpedia at https://dbpedia.org/page/X. The abundance of resulting RDFs for one query prevented us from exhibiting the concluding results for a sample query. Please note that first letter capitalization and white space replacement with underscore for multiword concepts are necessary.

**Step 2 .**

Knowledge Graph Enriching

After building the knowledge graph for the input text, different pieces of information (likewise in form of RDF triples) enrich the current knowledge graph during this step. Enriching the representation inevitably gives more focus to some neglected aspects of facts about entities. In other words, having limited aspects of knowledge will bound the intelligent agent's perception of the world [50]. Consequently, the following graph enrichments were carried out on the resulting knowledge graph.

*(i) Ontology-Based Enrichment*. Ontology actually is a branch of metaphysics dealing with the nature and relations of beings [51]. It demonstrates how the things are related to each other's in a systematic hierarchical classification.

A great deal of attention must be paid that knowledge bases essentially are made up of instances, rather than concepts; what are the foundations of ontologies? Therefore, it would indisputably enhance the representation by means of providing a different aspect of knowledge about things.

To do so, we used the DBpedia ontology. It covers 768 classes (a complete list of covered classes is available in [52]), which are described by 3,000 properties for about 4,233,000 instances. One can easily find the ontology-based representation of a given concept X in DBpedia ontology at https://dbpedia.org/ontology/X. At the beginning of foundation, it had been created based on most commonly used infoboxes within Wikipedia (in 2008) before it evolved into a crowd-sourcing effort. All the RDFs resulting from matching the concepts with DBpedia ontology were added to the previously achieved RDF graph.

*(ii) NRC Properties Enrichment*. Words can be associated with different intensities of an emotion. The NRC Emotion Intensity Lexicon [53], which is provided by National Research Council Canada (NRC), contains real-valued intensity scores for eight basic emotions (namely, anger, anticipation, disgust, fear, joy, sadness, surprise, and trust) for about 10,000 entries in English. The lexicon mainly includes more common English words and terms along with those that are more prevalent in social media. The aim of present section is to enrich the representation of input text through enhancing the eight provided emotions' degrees for included words. The emotions' scores for each concept were added to the existed RDF graph in the form of literal RDFs for each word, in case of inclusion in NRC.

*(iii) MRC Properties Enrichment*. The final knowledge graph enriching process was the enhancement of psycholinguistic properties to the RDF graph. It was carried out through MRC psycholinguistic database [54]. MRC is a publicly available machine useable dictionary, which contains (up to 26) linguistic and psycholinguistic attributes (like syntactic, phonological, orthographic, and semantic features) for 150,837 English words. These properties also were added to the existing RDF graph in the form of literal RDFs for each word.

**Step 3 .**

Knowledge Graph Embedding

So far, we have achieved the knowledge graph for a given text that basically is made up of RDFs, for both vertices and edges. This step transforms the resulting knowledge graph into a vector space and produces its equivalent embedding matrix. It strives to maximally persevere graph's structure, even though it practically performs dimensionality reduction on it. In this study, the knowledge graphs were embedded according to the method proposed by Ristoski et al. [55]. In their seminal contribution, they proposed RDF2vec, a tool for creating vector representations of RDF graphs. RDF2vec actually is inspired by the word2vec [56], which is a well-known word embedding method (representing words in numeric vector space). RDF2vec almost works similar to word2vec; the major difference is the input sequence. While word2vec receives a set of sentences for training the learning model as the input sequence, RDF2vec uses random walks on the RDF graph to create sequences of RDF vertices to feed them into the same learning model. As a consequence, similar vertices placed close to each other in the final vector space and dissimilar ones do not, like what happens for words after embedding in word2vec. To put it briefly, in this step, the corresponding embedding matrix for a given knowledge graph was achieved.

We set the maximum depth for each walk and the maximum number of walks per entity, both equal to 5 in all the random walks, which were carried out on knowledge graphs. It should be considered that, in practice, the two first phases, namely, preprocessing and knowledge representation, were iteratively executed for all of the essays in Essays Dataset (please refer to Figure 4) and lasted for more than four months. Experiments were run on a computer with an Intel i7-7700K processor, using 64 GB of ram and running Windows 10. As a result of such iteration, a set of embedding matrices resulting from the knowledge graphs embeddings were achieved, in which the rows of each matrix are dedicated to existing concepts in corresponding essay, and the columns are dedicated to the embedding dimensions. The number of rows in each matrix is different depending on the existing number of concepts in corresponding essay. Therefore, to fix the number of rows and achieve embedding matrices with same number of rows, we selected the 10,000 most frequent concepts in all final resulted knowledge graphs for Essays Dataset's essays. The larger number of rows leads to sparsity of embedding matrices, and the smaller number leads to ignore the included concepts. The number of columns (embedding size), which is specified by RDF2vec, by default is equal to 500.

In consequence, a brief summary of the phase 2, as it can be seen in Figure 4, may be described as follows:

- Input: a set of concepts for each text;
- Output: equivalent embedding matrix for each text;
- Objective: knowledge representation for each text; specifically building, enriching, and embedding the corresponding knowledge graph for each text.

#### 4.2.3. Phase 3: Automatic Personality Prediction

**Figure 5:**
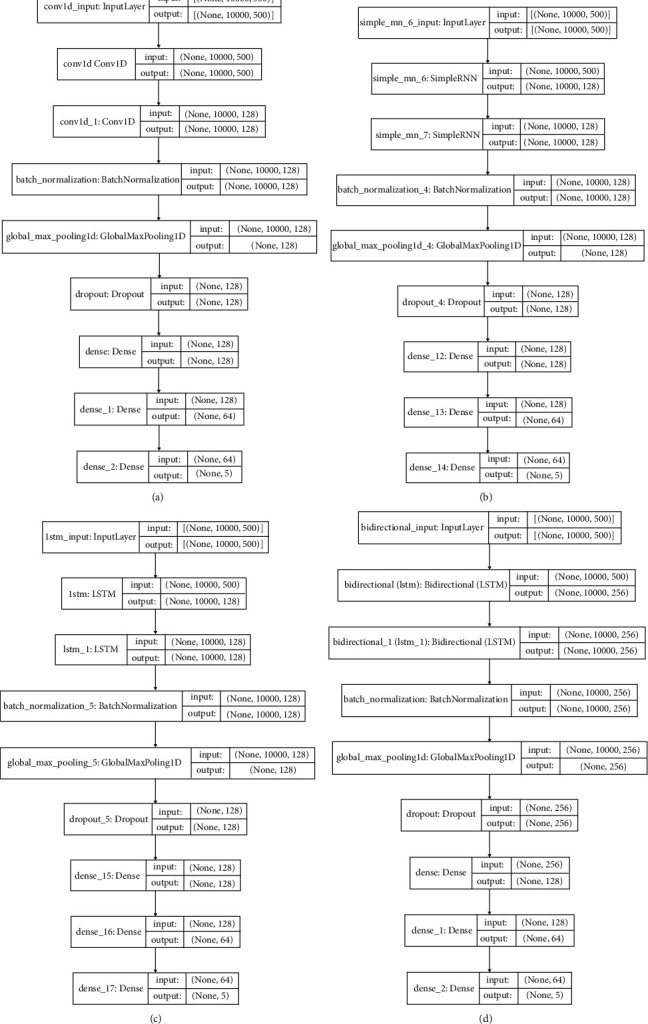
The Summaries of proposed APP classifiers. (a) CNN model. (b) RNN model. (c) LSTM model. (d) BiLSTM model.

Finally, four separate classification models were developed to carry out personality prediction, including convolutional neural network- (CNN-) based, simple recurrent neural network- (RNN-) based, long short-term memory- (LSTM-) based, and bidirectional LSTM- (BiLSTM-) based classifiers. To appraise the competency of suggested knowledge graph-enabled APP merely, some of the base and most well-known deep learning classification models, with maximally similar architectures and same configurations, were used. Classification in all Big Five traits was fulfilled concurrently. In fact, each model performs a multilabel binary classification, which assigns five labels to each of the OCEAN traits for a given text. Some common settings, which were applied in all suggested APP models, are presented in Table 1. Furthermore, as shown in Figure 5, the architecture of each model is composed of two stacked classifiers (like CNN), which leads to better results rather than single classifier. The classifiers are then followed by a batch normalization, to expedite the training and regularize the model. Next, applying a pooling layer as well as a dropout layer will help to avoid overfitting through providing an abstracted form of the representation. Finally, the models are followed by two consecutive dense layers to classify the extracted features from previous layers, change the dimensions of the vectors, and make possible the final prediction in the output layer.

*(i) Convolutional Neural Network- (CNN-) Based Classifier*. Convolutional neural networks, as a model with impressive performance, have been extensively investigated in various problems including visual recognition, speech recognition, and natural language processing [57]. To classify the resulted embedding matrices, a model with two one-dimensional convolutional layers followed by a batch normalization layer, a pooling layer, a dropout layer for regularization, and finally two fully connected layers was developed. In each convolutional layer, 128 parallel feature maps and a kernel size of 7, along with same padding, were applied.  Figure 5(a) presents a summary of the model.

*(ii) Recurrent Neural Network- (RNN-) Based Classifier*. Recurrent neural networks have shown excellent dexterity in text classification tasks. The foundation of RNN [58] makes it possible to utilize previous step's outputs as inputs in current step. To rephrase it, while traditional neural networks deal with the inputs independently of one another, RNNs manipulate a set of previous inputs. Furthermore, the internal state of an RNN, which acts as memory, empowers it to learn from previous information and grants a privilege of processing the sequential inputs like text. The suggested simple RNN-based classifier encompasses two simple RNN layers followed by a batch normalization layer, a pooling layer, a dropout layer for regularization, and finally two fully connected layers. A summary of the model is shown in Figure 5(b).

*(iii) Long Short-Term Memory- (LSTM-) based classifier*. Long short-term memory networks as a kind of RNNs are suggested to deal with learning long-term dependencies problem [58]. In simple terms, simple RNNs suffer one major drawback; they can not remember information for a long period of time, what is resolved capably by LSTMs. Two stacked LSTMs followed by a batch normalization layer, a pooling layer, a dropout layer for regularization, and finally two fully connected layers construct the design of proposed LSTM-based classification model. A plot of model is depicted in Figure 5(c).

*(iv) Bidirectional Long Short-Term Memory- (BiLSTM-) based classifier*. Indeed, as can be inferred, BiLSTMs are a bidirectional form of LSTMs. In simple words, LSTM is a unidirectional network, which utilizes previous information that has already passed through it in forward direction within sequence processing, while a BiLSTM network exploits both previous and future information in forward and backward directions, respectively. Telling the truth, it consists of two LSTMs: one analyzes the input sequence from beginning to the end in forward direction, and the other one, from end to beginning in backward direction [59]. The final output is the concatenation of the two LSTMs. Two stacked BiLSTMs, followed by a batch normalization layer, a pooling layer, a dropout layer, and finally two fully connected layers, comprise the architecture of the proposed BiLSTM-based classifier.  Figure 5(d) depicts a summary of the model.

At last, a brief outline of phase 3 as can be seen in Figure 4 is as follows:

- Input: a set of embedding matrices;
- Output: predicted labels for OCEAN traits for each embedding matrices;
- Objective: personality prediction using multilabel classification model.

Algorithm 1 details a step-by-step flow of the proposed method that would assist towards a better comprehension of the method.

## 5. Results

### 5.1. Evaluation Metrics

Traditionally, classification models are evaluated through some well-known evaluation metrics including precision, recall, f-measure, and accuracy [39]. There are two determining sets, which play crucial role in their values, specifically the set of essays' “actual labels,” which is sometimes referred to as gold standard and the set of “system predicted labels.” Practically, for each prediction in a given class (namely, O, C, E, A, and N), there are four possible combinations of actual labels and system predicted labels, including:

- True Positive (TP): that occurs when the actual label is true, and the system predicted label is also true;
- True Negative (TN): that occurs when the actual label is false, and the system predicted label is also false;
- False Positive (FP): that occurs when the actual label is false, while the system predicted label is true;
- False Negative (FN): that occurs when the actual label is true, while the system predicted label is false.

Essentially, in an APP system evaluation, the TP and TN play a leading part, due to the fact that, in such classification systems, it is prominent to truly predict that a given text really belongs or does not belong to the class. In both of TP and TN, the system predicted labels are equal to the actual labels; hence, it can be stated that the total number of TPs and TNs denotes the APP system's correct predictions. Consequently, the ratio of systems correct predictions to the total number of predictions reveals the quality of prediction; it is actually known as accuracy. That is to say, accuracy=(TP+TN)/(TP+TN+FP+FN).

Moreover, the precision and recall as well as their weighted harmonic mean, which are called f-measure, convey some facts about the performance of classification system. Precision (*P*) mainly concerns system's true labeled predictions. It reveals that the proportion of system's true labeled predictions has actual true labels. In other words, *P*=TP/(TP+FP), while recall (*R*) mainly concerns true labels in gold standard. It tries to reveal that proportion of true labeled samples in gold standard has achieved true labels, after the system prediction. It means that *R*=TP/(TP+FN).

Both of the precision and recall are unreliable metrics in classification systems' evaluation when they are considered separately. To put it another way, there may be some cases with high values of precision and low values of recall simultaneously, and vice versa. It is principally because of their partial coverage and incomplete reports. Hence, f-measure is suggested to address this problem. In fact, it makes a tradeoff between precision and recall and combines their included facts; precisely, *f* − measure=(2 × *P* × *R*)/(*P*+*R*). However, it still suffers from a significant drawback. Actually, TN as a prominent factor in evaluation is completely neglected. As an illustration, it ignores all of the correctly false labeled samples by system. Thereby, accuracy is preferred to f-measure in APP system evaluation.

### 5.2. Evaluation Results

**Figure 6:**
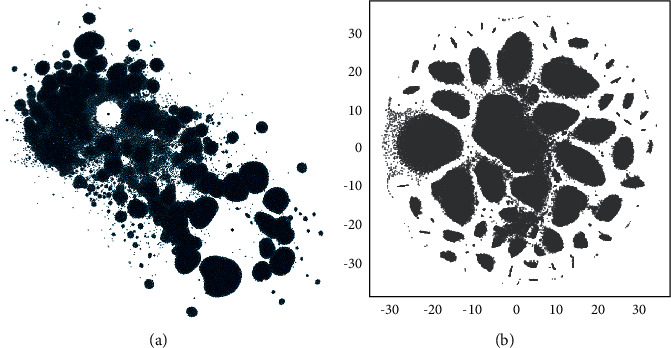
The resulting final knowledge graph from phase 2 for a sample essay (2004_139) in Essays Dataset (including 226,763 vertices and 532,146 edges). The edges and labels in knowledge graph are emitted for better visualization. (a) Knowledge graph's vertices (provided by Gephi, the ForceAtlas2 algorithm). (b) Embedded knowledge graph in 2D space.

This study was undertaken to design a knowledge graph-enabled automatic personality prediction system and evaluate the efficacy of knowledge graph-enabling of a personality prediction system. Accordingly, a three-phase approach was proposed, which by receiving a text proceeds to carry out some preprocessing in the first phase and then build, enrich, and embed the corresponding knowledge graph consecutively in second phase, as it is completely scrutinized in Section 4.2.2.  Figure 6 provides the results obtained from the second phase for a sample essay in Essays Dataset. Eventually, the resulting embedding matrix was classified through four independent classification models in third phase, and the predicted labels in each OCEAN traits were assigned. This section summarizes the findings and contributions made.

Specifically, there were four APP classification models suggested, namely, CNN-based, RNN-based, LSTM-based, and BiLSTM-based classifiers. However, accuracy outperforms precision, recall, and f-measure in APP systems' evaluation, and we will report the evaluation results for all of them. Albeit that, we will mainly rely on accuracy. Of course, in spite of the facts behind the precision, recall, and f-measure, availability of their values would be helpful when comparing those studies, which have just reported the evaluation results for them, rather than accuracy.

**Figure 7:**
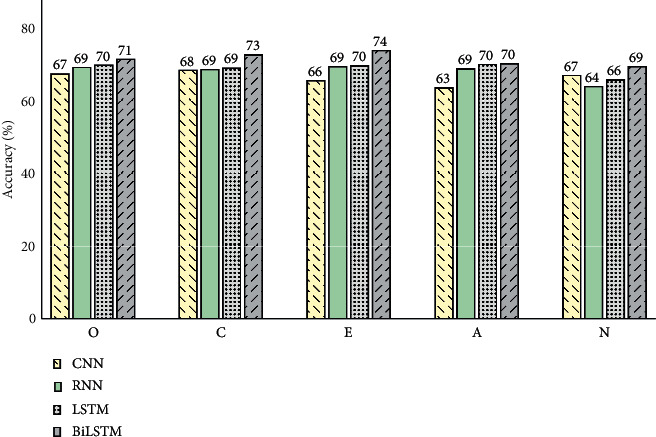
Accuracy values for four suggested APP classifiers, in each of the five personality traits in Big Five model (results are rounded).

Table 2 provides the results obtained from the evaluation of four APP classifiers. Comparing accuracy values among four suggested classifiers, the most striking results were achieved through BiLSTM. Specifically, it had the most accurate predictions in all OCEAN traits compared to other classifiers. Hence, the first highest average accuracy in five traits was achieved by BiLSTM. In addition, the second highest average accuracy was attained by LSTM. However, comparing the accuracies in each trait individually reveals that it had more accurate predictions in *O*, *C*, *E*, and *A* rather than RNN and CNN, while CNN in *N* practically had better predictions. However, LSTM concluded more accurate results rather than simple RNN in *N*. Afterwards, RNN outperformed CNN in all traits except *N*.  Figure 7 compares the accuracy values in five personality traits resulting from four classification models.

As Table 2 shows, the same ranking as it happens when perusing the accuracy was achieved by classification models when taking the average f-measure into consideration. That is to say, BiLSTM, LSTM, RNN, and at last CNN were ranked first to fourth, respectively, although the ranks do not last when comparing the f-measure values individually in each trait. Regarding recall average values, LSTM with a slight difference to BiLSTM and CNN had better performance. At last, among the four suggested classifiers, BiLSTM, LSTM, RNN, and CNN had the most precise predictions, respectively, as it can be seen from Table 2.

## 6. Discussion

The major objective of current study was to investigate the efficacy of knowledge graph-enabled automatic personality prediction system. Thus, we used four simple base deep learning classifiers, which were designed maximally similar to each other. Furthermore, we intentionally avoided designing complex networks to merely appraise the efficacy of knowledge graph-enabling of an APP system. Accordingly, we suggested one CNN-based classifier as well as three recurrent classifiers, in particular, a simple RNN-based classifier along with one LTSM-based and one bidirectional LSTM- (BiLSTM-) based classifiers.

Regarding the resulting accuracy values in Table 2 for each of the classifiers, it is clear that generally recurrent classifiers lead to better results, rather than CNN. It seems possible that these results are due to their ability in processing temporal information that is presented in input sequences. To put it simply, recurrent networks are basically designed for sequence prediction problems like text. More specifically, they can capture sequential information, which pinpoints the existing dependencies among the words throughout the input sequence of words.

Among the three suggested recurrent networks, superior results are seen for BiLSTM. In fact, it outperforms LSTM and simple RNN, in which this does seem to be because it is actually an enhanced version of LSTM, in which it itself is an enhanced version of simple RNN. To rephrase it, LSTMs were proposed to tackle RNNs' problem in preserving information over several timesteps; and BiLSTMs were also proposed to tackle LSTMs' problem in ignoring future information for a given word in input sequence. As a consequence, it is fair that BiLSTMs show better results than LSTMs and simple RNNs, and LSTMs show better results than simple RNNs. This is what happened in all personality traits, though with slight differences in some traits. So, the obtained results confirm the expectations.

Besides, CNN-based classifier leads to comparable results. Despite the fact that it was ranked fourth among four classifiers, its results are so close to some recurrent classifiers in *C* and even it outperforms simple RNN and LSTM-based classifiers in *N*. We speculate that this might be due to its filters' good ability in feature extraction from input embedding matrices.

The results obtained from the four proposed classifiers can be compared with the state-of-the-art APP systems, which were performed on Essays Dataset in Table 3. These results go beyond previous contributions, showing that all of the suggested methods give clearly better results than all of them. On the other hand, whereas our first ranked proposed method considerably yields better results, the fourth ranked proposed method also outperforms that of previous reports. This is an important finding in the understanding of the knowledge graph-enabling of an automatic personality prediction system. Moreover, it is anticipated that utilization of more complex classification models (like hybrid models) would lead to more accurate predictions.

Ultimately, we are going to answer the research questions (as stated in Introduction) according to our observations as follows:

  RQ.1: The results of the experiment found clear support for knowledge graph-enabling of an APP system. Actually, it empowers an APP system to yield considerably more accurate results. It is also worth noting that, in this study, we have just utilized the embeddings of resulting knowledge graphs to perform personality predictions, while the knowledge graphs inherently comprise miscellaneous knowledges of concepts, which may be effectively utilizable in automatic personality prediction.
  RQ.2: The most interesting finding was that, in classification of knowledge graphs' embedding matrices, all of the proposed deep learning classifiers, namely, CNN-based, RNN-based, LSTM-based, and BiLSTM-based classifiers, substantially outperform the state-of-the-art contributions in APP, in spite of the models' simple design. This is obviously confirmed when comparing our results to those of older studies. Besides, experimental observations demonstrated that the classifiers, which are based on BiLSTM, LSTM, simple RNN, and CNN, yield better results, respectively, when they were utilized in classification of knowledge graphs' embeddings.
  RQ.3: Regarding the obtained results from several classifiers, it is clear that knowledge graph-enabling of an APP system totally enhances the number of accurate predictions in all personality traits of Big Five model, albeit the enhancement pattern is not similar in all of the classifiers.

## 7. Conclusion

The current study aimed to determine the effect of knowledge graph-enabling on an automatic personality prediction system. To do so, a three-phase approach was proposed, in which a given text performs some preprocessing (including tokenization, noise removal, normalization, and named entity recognition) in its first phase. The second phase is aimed toward achieving a knowledgeable representation of input text, trying to build the corresponding knowledge graph, then enriching it (utilizing DBpedia ontology, NRC Emotion Intensity Lexicon, and MRC psycholinguistic database), and finally embedding the enriched knowledge graph. At last, in the third phase, the embedding knowledge graph is fed into some base deep learning models (namely, CNN-based, simple RNN-based, LSTM-based, and BiLSTM-based classifiers) to perform personality prediction. The results demonstrate a strong effect of knowledge graph-enabling on an automatic personality prediction system. More specifically, the findings definitely confirmed the proposed method's ability to predict all five personality traits of the Big Five model.

As the greatest practical significance of this study, it provides the basis of human-like behavior for machines in a specific task, namely, automatic personality prediction. Since human intelligent behavior is a consequence of his/her cognitive abilities, in which it is an outcome of representation of the knowledge of the world's concepts; therefore, our method will help machines mimic human behavior as it is, which is a big step forward. That is to say, providing a comprehensive representation of appearing concepts in the input text models the human cognition for the machine, which enables it to show human-like performance. The obtained results, as well as the comparison of the findings with those of other studies, confirmed this claim.

In future work, we intend to investigate more complex deep learning models, to achieve more accurate predictions. The current study has only examined the efficacy of a knowledge graph-enabled automatic personality prediction system, and hence to minimize the effect of extrinsic factors as far as possible, it just relied on simple base deep learning models. As well, since the resulted knowledge graph usually is very large, more research is also needed to find a way to cope with it. Besides, further experimental investigations are needed to peruse other graph embedding methods and determine their effectiveness. Moreover, the application of the suggested method over different datasets in different personality models could shed more light on the efficacy of the proposed method. More broadly, the proposed knowledge representation method potentially is capable of performing other tasks, which deal with text, since it provides a more knowledgeable representation of text elements for machines. Hence, the issue of knowledge representation is an intriguing one, which could be usefully explored in several researches.

## Figures and Tables

**Algorithm 1:**
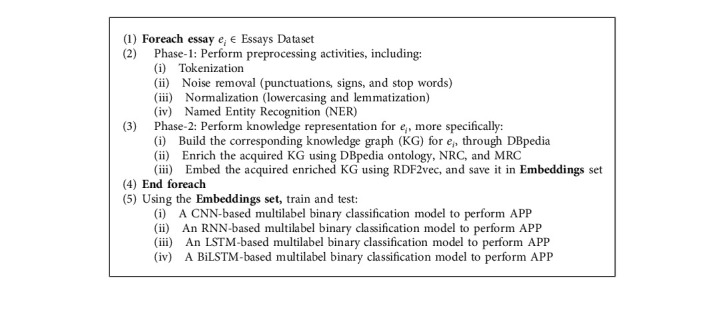
Algorithm of the proposed method

**Table 1:** Common parameters' settings among all of the proposed APP models (including CNN, RNN, LSTM, and BiLSTM).

Parameter	Setting	Parameter	Setting
Train-test split ratio (%)	80–20	Optimizer	Stochastic gradient descent (SGD)
Number of epochs	30	Learning rate	0.01
Early stopping	Applied on validation loss	Loss function	Binary_cross-entropy
Patience value	4	Batch size	32
Activation function	Sigmoid	Cross validation	10-Fold

**Table 2:** Evaluation results for suggested APP classifiers, including CNN-based, RNN-based, LSTM-based, and BiLSTM-based classifiers.

Metric	Classification model	O	C	E	A	N	Avg.
Precision	CNN	61.22	60.70	60.13	59.80	62.42	60.85
	RNN	62.41	62.80	64.49	61.63	55.52	61.37
	LSTM	66.56	67.65	63.79	67.58	59.62	65.04
	BiLSTM	69.12	71.43	73.05	67.75	62.69	68.81

Recall	CNN	82.68	79.72	78.36	74.37	78.48	78.72
	RNN	77.73	71.69	77.06	74.30	76.67	75.49
	LSTM	81.20	79.31	82.76	78.88	71.82	78.79
	BiLSTM	78.80	80.46	83.03	76.33	75.12	78.75

*F*-measure	CNN	70.35	68.92	68.05	66.29	69.53	68.63
	RNN	69.23	66.95	70.22	67.37	64.40	67.64
	LSTM	73.15	73.02	72.05	72.79	65.15	71.23
	BiLSTM	73.64	75.68	77.72	71.78	68.34	73.43

Accuracy	CNN	67.34	68.36	65.52	63.49	66.94	66.33
	RNN	69.17	68.56	69.37	68.76	63.90	67.95
	LSTM	69.78	68.97	69.57	69.98	65.72	68.44
	BiLSTM	71.40	72.62	73.83	70.18	69.37	71.48

**Table 3:** Comparing the results obtained from our proposed methods and state-of-the-art reports in APP from text, which were performed on Essays Dataset.

APP method	F-measure						Accuracy					
	O	C	E	A	N	Avg.	O	C	E	A	N	Avg.
CNN-based classifier (proposed method)	70.35	68.92	68.05	66.29	**69.53**	68.63	67.34	68.36	65.52	63.49	66.94	66.33
RNN-based classifier (proposed method)	69.23	66.95	70.22	67.37	64.40	67.64	69.17	68.56	69.37	68.76	63.90	67.95
LSTM-based classifier (proposed method)	73.15	73.02	72.05	**72.79**	65.15	71.23	69.78	68.97	69.57	69.98	65.72	68.44
BiLSTM-based classifier (proposed method)	**73.64**	**75.68**	**77.72**	71.78	68.34	**73.43**	**71.40**	**72.62**	**73.83**	**70.18**	**69.37**	**71.48**
Majumder et al. [6]							62.68	57.30	58.09	56.71	59.38	58.83
Yuan et al. [9]							62.00	57.00	58.00	56.00	59.00	58.40
Ramezani et al. [24]	57.37	59.74	65.80	61.62	60.69	61.04	56.30	59.18	64.25	60.31	61.14	60.24
Xue et al. [28]	67.84	63.46	71.50	71.92	62.36	67.42	63.16	57.49	58.91	57.49	59.51	59.31
El-Demerdash et al. [30]							63.30	57.97	58.85	59.25	59.88	59.85
Jiang et al. [31]							65.86	58.55	60.62	59.72	61.04	61.16
El-Demerdash et al. [13]							65.60	59.52	61.15	60.80	62.20	61.85
Kazameini et al. [34]							62.09	57.84	59.30	56.52	59.39	59.03
Wang et al. [60]	67.00	68.00	67.00	69.00	69.00	68.00	64.80	59.10	60.00	57.70	63.00	60.92
Tighe et al. [61]	61.90	56.00	55.60	55.70	58.30	57.50	61.95	56.04	55.75	57.54	58.31	57.92

## Data Availability

The data used to support the findings of this study are available from the corresponding author upon request.
